# 

**Published:** 1913-09-20

**Authors:** 


### HOSPITAL BEDSTEADS.

At the International Medical Congress J. Nesbit-Evans and Co., of Floodgate Street, Birmingham, have been awarded a gold medal and diploma for their "Nesbit-Evans" Hospital Bedsteads by the special International Award Committee, which included various eminent British, Colonial, American, and other foreign surgeons and hospital administrators. The particular beds which secured the award were the patent "Balcony" Wheeling-out General Hospital Bedstead and the new Surgical Bedstead having patent Bed-inclining Stand, which dispenses with the old unsafe wooden blocks as in use in various hospitals.

### Gifts and Bequests.

Mr. William Dunmore has endowed a cot, in memory of "a noble wife and mother," with the sum of £500, at Leicester Royal Infirmary and Children's Hospital.

Mr. Arthur Jenkins Harrop, of Stockport, retired bank manager, has left £500 each to Dr. Barnardo's Homes and the Stockport Infirmary; £300 to the Stockport Sick Poor Nursing Association; and £200 to the Stockport Institution for the Blind, Deaf, and Dumb.

Mr. Benjamin Evans, of Carmarthen, has bequeathed £900 each to the Swansea Hospital and George Muller's Orphanage, Ashley Down, Bristol; £225 each to the Deaf and Dumb Institution, Swansea, and the Institution for the Blind, Swansea; and £100 each to the Earlswood Asylum and the Swansea Orphan Home.

Mr. Harry Smallwood, of Birmingham, has left the ultimate residue of his estate upon trust for his children or their issue in equal shares. Failing issue, the testator left £1,250 to the General Hospital, Birmingham, and £1,000 each to the Birmingham and Midland Hospital for Women and the Birmingham and Midland Hospital for Sick Children. The ultimate remainder of his estate he bequeathed to the General Institution for the Blind, Edgbaston, and the Royal Institution for Deaf and Dumb Children, Birmingham.

### Sir William Dunn's Hospital Bequests.

Under the will of the late Sir William Dunn, Bart., many important hospital bequests are made. He left £2,000 (£500) to the Charity Organisation Society, £5,000 (£500) to the Royal Caledonian Asylum, £3,000 (£500) to Dr. Barnardo's Homes, £5,000 (£500) to the Homes for Little Boys, £5,000 (£500) to the Reedham Orphanage, £5,000 (£500) to the Royal Merchant Seamen's Orphanage, £2,000 (£500) to the London Orphan Asylum, Watford, £2,000 (£1,000) to King Edward's Hospital Fund for London, £5,000 (£500) to the Free Cancer Hospital, Fulham Road, S.W., £5,000 (£500) to the Hospital for Consumption and Diseases of the Chest, Brompton, £5,000 (£1,000) to the London Hospital, £2,000 (£1,000) to the Middlesex Hospital for the Cancer Ward, £2,000 (£1,000) to the Hampstead General Hospital, £2,000 (£1,000) to the Royal Eye Hospital, Southwark, S.E., £2,000 (£1,000) to the Mount Vernon Hospital for Consumption and Diseases of the Chest, £3,000 (£1,000) to the Royal Hospital for Incurables, Putney, £1,000 to the British Home and Hospital for Incurables, Streatham, £2,000 (£1,000) to the National Industrial Home for Crippled Boys, Kensington, £3,000 to Mrs. Gladstone's Convalescent Free Home for the Poor, Mitcham (altered in pencil to £1,000), £2,000 (£1,000) to the East End Emigration Fund, Stepney, £2,000 (£1,000) to the West London Hospital for Children, Chelsea, £1,000 to the Victoria Hospital for Children, Chelsea, £1,000 to the West End Hospital for Nervous Diseases, Welbeck Street, W., £2,000 to the Royal National Hospital for Consumption, Ventnor, £1,000 (cancelled in pencil) to the General Lying-in Hospital, York Road, S.E., £2,000 (£1,000) to the Institute of Medical Sciences, University of London, £10,000 (£2,000) to trustees for division between the Orphan Homes of Scotland, the Consumption Sanatorium of Scotland, and the Colony of Mercy for Epileptics (Quarrier's), £2,000 (£1,000) to the Paisley Society for the Treatment of Diseases considered incurable, £2,000 (£1,000) to the Paisley Female Refuge, £1,000 to the Paisley and District Mission to the Deaf and Dumb, £1,000 to the Royal Victoria Eye Infirmary, Paisley, £1,000 (£500) to the Paisley Convalescent Home, West Kilbride.

It is noteworthy that according to an affidavit which was filed with the will many pencil alterations were made after the will's execution. It is presumed that as these are not attested in any way the bequests as originally written stand good, but in the above list of bequests the amount as originally written is given first, followed by the pencil alteration in parentheses. The legacies to his family are to be paid first, then those to employees, then those for public uses under £2,000 in amount, then those of from £2,000 to £5,000, and then those of over £5,000. The residue of his property he left to his trustees for such of the above-mentioned charitable purposes (except the purchase of a park for the West End of Paisley) as his trustees may think fit.

### Appointments.

Dr. T. C. Ritchie and Dr. W. W. MacNaught have been appointed indoor house surgeons to the Glasgow Maternity and Women's Hospital for six months from October 6, and Dr. David W. Reid and Dr. James Angus outdoor house surgeons to the hospital for three months from the same date.

Professor Francis Gotch, F.R.S., Waynflete Professor of Physiology at Oxford and formerly Holt Professor of Physiology at Liverpool, has left estate of the gross value of £19,928, of which £18,621 is net personalty. Sir Victor Horsley is one of the executors.

### Fellows' Syrup of the Hypophosphites

The uniformly high character of this preparation is steadfastly guaranteed by the manufacturers

Reject < Cheap and Inefficient Substitutes / Preparations "Just as Good"

### HANDBOOKS FOR TRAINED WORKERS

Bound in limp cloth.

1/- net.

1/1 post free.

POCKET-GUIDE SP SERIES

Suitable size for the pocket.

1/- net.

1/1 post free.

The Works which comprise the S.P. Pocket Guide Series are intended for ready reference, consequently the aim of the Publishers has been to make them as concise and lucid as possible. Each book is written by an expert on the subject of which it treats, and the utmost reliance therefore can be placed in the information it imparts.

**Essentials of Fever Nursing.** By Lytton Maitland, M.D. (Lond.), M.B., B.S., D.P.H. (Camb.).
**Manual and Atlas of Swedish Exercises.** (*With over 60 Illustrations.*) By Thomas D. Luke, M.D., F.R.C.S.
**Bandaging Made Easy.** (*Illustrated with over 90 Diagrams.*) By M. Hosking, Sister-in-Charge, Tredegar House.
**How to Write and Read Prescriptions.** By Lytton Maitland, M.D. (Lond.), M.B., B.S., D.P.H. (Camb.).
**Principal Drugs and their Uses.** By A Pharmacist.
**Treatment after Operations.** *Rules for Nursing after General and Special Operations.* By Mary Wiles.
**The Nurse's Duties Before and During Operations.** By Margaret Fox, Matron, Prince of Wales's General Hospital, Tottenham.
**Asepsis and How to Secure It.** By H. W. Carson, F.R.C.S. [*In the Press.*

***Other Works, in amplification of this Series, will be announced in due course.***

*Published by*
**THE SCIENTIFIC PRESS, Ltd.,**
**28/29 Southampton Street, Strand, London, W.C.**

